# Processing of Fear and Anger Facial Expressions: The Role of Spatial Frequency

**DOI:** 10.3389/fpsyg.2013.00213

**Published:** 2013-04-26

**Authors:** William E. Comfort, Meng Wang, Christopher P. Benton, Yossi Zana

**Affiliations:** ^1^Centro de Matemática, Computação e Cognição, Universidade Federal do ABCSanto André, Brazil; ^2^Institute of Neuroscience, Guangzhou Medical UniversityGuangzhou, China; ^3^School of Experimental Psychology, University of BristolBristol, UK

**Keywords:** spatial frequency, face adaptation, fear, anger, facial expressions, face processing

## Abstract

Spatial frequency (SF) components encode a portion of the affective value expressed in face images. The aim of this study was to estimate the relative weight of specific frequency spectrum bandwidth on the discrimination of anger and fear facial expressions. The general paradigm was a classification of the expression of faces morphed at varying proportions between anger and fear images in which SF adaptation and SF subtraction are expected to shift classification of facial emotion. A series of three experiments was conducted. In Experiment 1 subjects classified morphed face images that were unfiltered or filtered to remove either low (<8 cycles/face), middle (12–28 cycles/face), or high (>32 cycles/face) SF components. In Experiment 2 subjects were adapted to unfiltered or filtered prototypical (non-morphed) fear face images and subsequently classified morphed face images. In Experiment 3 subjects were adapted to unfiltered or filtered prototypical fear face images with the phase component randomized before classifying morphed face images. Removing mid frequency components from the target images shifted classification toward fear. The same shift was observed under adaptation condition to unfiltered and low- and middle-range filtered fear images. However, when the phase spectrum of the same adaptation stimuli was randomized, no adaptation effect was observed. These results suggest that medium SF components support the perception of fear more than anger at both low and high level of processing. They also suggest that the effect at high-level processing stage is related more to high-level featural and/or configural information than to the low-level frequency spectrum.

## Introduction

Face perception includes the integration of complex visual input across a spectrum of varying spatial frequencies (SFs) (Sowden and Schyns, 2006). Different SFs are transmitted to higher cortical regions through separate neurological channels broadly related to the magnocellular and parvocellular pathways originating in the retina. High spatial frequency (HSF) information is processed primarily through the ventral visual processing stream from V1 and encodes the fine-grained texture of objects such as faces. In contrast, low spatial frequency (LSF) information is encoded through faster, more direct networks in subcortical and early visual areas and communicates rapid, coarse signals concerning the configuration or spatial relationship between facial features (for an overview, see Ruiz-Soler and Beltran, 2006). Subsequent outputs from these pathways typically converge in higher-level regions, including the amygdala, fusiform gyrus, and orbitofrontal cortex (Vuilleumier et al., 2003; Bar et al., 2006; Rotshtein et al., 2007). Previous studies have suggested that information from high and low SF bandwidths may be effectively integrated at these sites, producing a neural representation of the face to guide subsequent recognition (Eger et al., 2004; Gauthier et al., 2005; Rotshtein et al., 2007). While the integration of facial identity through SF information has been well documented (Costen et al., 1996; Näsänen, 1999; Goffaux et al., 2003; Rotshtein et al., 2007; Gao and Bentin, 2011), there is less information about how our neural representation of a facial emotion is related to specific SF ranges. Studies which have focused on this issue (Vuilleumier et al., 2003; Deruelle and Fagot, 2004; Aguado et al., 2010; Kumar and Srinivasan, 2011) have examined the effect of high and low SF on the perception of emotional expressions using removal of specific SF components from the target stimuli. For example, Kumar and Srinivasan (2011) showed that low (<8 cycles/face) and high-frequencies (>32 cycles/face) are more critical for the representation of happy and sad expressions, respectively. Aguado et al. (2010) compared reaction time to classification of happy and anger faces and found an advantage of LSF (<12 cycles/degree) over HSF (>3 cycle/degree). Vuilleumier et al. (2003), on the other hand, found no differences between reaction time and accuracy for discrimination of fear over neutral expression for low (2–8 cycles/face) and middle (8–16 cycles/face) SF band-pass filtered images. Similarly, Deruelle and Fagot (2004) found no differences in adult subjects between discrimination of low (<12) and high-pass (>36) filtered faces with a smiling or grimacing expression (participants aged below nine showed a bias toward high-pass filtered images). Although important, none of these results advance our current knowledge regarding the SF tuning at different phases of facial expression processing. In this study we are aim to discriminate between low and high levels of processing SF content in the representation of facial expressions. Potentially, this will reveal further details of how and at what stage differing SF bandwidths are integrated. Visual adaptation, a psychophysical method commonly associated with higher-order visual processing (Fox and Barton, 2007; Webster and MacLeod, 2011) may provide answers as to how visual SFs are processed at relatively late stages of facial expression analysis.

Visual adaptation, in the context of face processing, takes the form of prolonged exposure to a target face which can affect the subsequent perception of facial attributes and provide valuable insights into the pattern of visual coding used in facial processing (for an overview, see Webster and MacLeod, 2011). In a typical experimental setting, a subject is exposed to a particular face, causing faces which are presented afterward to be perceived as less similar to the adapting face in comparison to a condition with no adaptation. Such face adaptation aftereffects are robust across changes in the size, retinal position, and orientation of facial images and exhibit similar patterns of adaptation across low-level features such as contrast, color, and SF (Leopold et al., 2001; Watson and Clifford, 2003; Yamashita et al., 2005). Adaptation is also specific to facial attributes: aftereffects have been documented for facial properties such as identity (Leopold et al., 2001, 2005), gender, race, and expression (Webster et al., 2004; Furl et al., 2007). As these adaptation effects are uninfluenced by variations in low-level visual features (Webster and MacLeod, 2011), it is reasonable to assume they are associated with higher-level processing (Clifford et al., 2007). Electrophysiological evidence (Kovács et al., 2007) supports this hypothesis: ERP responses associated with facial aftereffects were more correlated with detailed encoding of the facial attribute rather than processing of low-level visual features.

As facial stimuli filtered to include only specific SF bandwidths have been shown to selectively activate higher-level areas associated with the semantic/functional content of faces (Vuilleumier et al., 2003; Rotshtein et al., 2007), the adaptation effects associated with certain facial stimuli are expected to vary according to their SF content. Yamashita et al. (2005) found that featural distortions in one SF range (e.g., LSF) of a facial stimulus failed to produce subsequent aftereffects in its opposing SF range (e.g., HSF), showing less transfer of adaptation effects across SF than other visual features such as image size. In addition, pairs of faces varying solely by SF content were rated as less similar than those differing according to other low-level features such as size, contrast, and color. Thus, SF information manipulation appears to drive face adaptation effects at a relatively high-level of visual processing. It should also be noted that spatially filtering a facial image may alter configural and featural properties of the face which are important for facial representation (Goffaux et al., 2005; Yamashita et al., 2005).

Clearly, evidence from face adaptation can offer key insights into how facial expressions are represented in the brain. Two alternative views predominate in this domain: one that posits that facial emotions are represented as discrete categories, with expression aftereffects serving to highlight the differential relationships between emotion categories (e.g., Rutherford et al., 2008). The other views face adaptation as equivalent to a shift in the prototype of a multidimensional “face space,” where the facial attribute is encoded in terms of its deviation from an average (e.g., Robbins et al., 2007). In the present study the latter continuum-based approach was taken.

Given a reference point *x_i_* at which a certain morphed face image is perceived as expressing fear and anger at equal probability (a balance point), manipulation of frequency content and adaptation state is expected to shift that point to one of the two extremes. For example, if low frequency components are used to encode fear more than anger, removing low SF content from the stimuli will increase the probability of the face image at point *x_i_* to be classified as expressing anger. Fear and anger expressions were chosen due to the fact that these form opposites along such a continuum in the feature dimension (e.g., distance between eyebrows and eyes, aperture of mouth) and they are negatively correlated in terms of their diagnostic information (Smith et al., 2005) and evolutionary perspective (anger as dominant, fear as submissive; see Leppänen and Nelson, 2009). Adaptation to either facial expression is expected to cause the morphed face to appear more similar to the opposite expression. As an alternative to methods adopted by other studies examining adaptation to facial expressions (Hsu and Young, 2004; Fox and Barton, 2007; Juricevic and Webster, 2012), this design evaluates adaptation aftereffects in the context of ambivalent expressions instead of neutral expressions (Webster et al., 2004).

Experiment 1 looks at the classification of emotional expressions under conditions of limited SF information; specifically, when components of high, medium, or low frequency are missing from the target face image. This method probes the use of specific SF information for encoding facial expression at a low and/or high level of processing, and was used to determine what SF range will be likely recruited in later adaptation. In Experiment 2 we tested the combined adaptation effect of magnitude and phase components on classification of morphed facial expressions of fear and anger, through adaptation to filtered and unfiltered images of prototypical fear. In Experiment 3 filtered and unfiltered adaptation stimuli were constructed and presented such that only the amplitude spectrum from a specific spectral bandwidth was retained, while its corresponding phase spectrum was randomized. Thus, we tested the working hypothesis that fear and anger expression processing is tuned to specific SF bandwidths, independent of the featural or configural information in the face. By comparison of the results from Experiment 2 and 3, we can estimate the relative use of SF content for high-level representation of facial expressions along a fear-anger axis. The results of this study demonstrate for the first time, to the best of our knowledge, differential SF tuning of facial fear and anger expression at low and high visual processing levels through behavioral paradigms.

## Experiment 1: Removing Specific SF Bandwidths from Test Stimuli

### Materials and methods

#### Participants and apparatus

Seven volunteers from the Universidade Federal do ABC (four male and four female between 18 and 37 years of age) with normal or corrected-to-normal vision participated in the experiment. Images were displayed on a Samsung SyncMaster 997MB monitor with screen dimensions of 368 × 276 mm, 1024 × 768 resolution, and refresh rate of 100 Hz; Gamma was corrected to produce a linear luminance-modulated image using a photometer (Tektronix, model J18, sensor model J1803) for luminance measurements. The monitor was driven by a Pentium D 3.40 GHz PC. PsychToolBox software (Pelli, 1997) was used to display all images in MATLAB^®^.

#### Stimuli

A total of 50 images with equal numbers of male and female faces were drawn from the Karolinska Directed Emotional Faces (KDEF) database (Lundqvist et al., 1998) to form a subset of 25 male and female images representing fear and a subset of 25 male and female images representing anger. The emotional content of the images were rated on intensity and arousal scales and it was shown that they comprise a valid set of affective facial pictures (Goeleven et al., 2008). Each of these subsets was gray-scaled and averaged across gender and identity to produce a prototypical fearful and angry face, measuring 512 × 512 pixels, using the methods described in Tiddeman et al. (2001).

**Figure 1:**
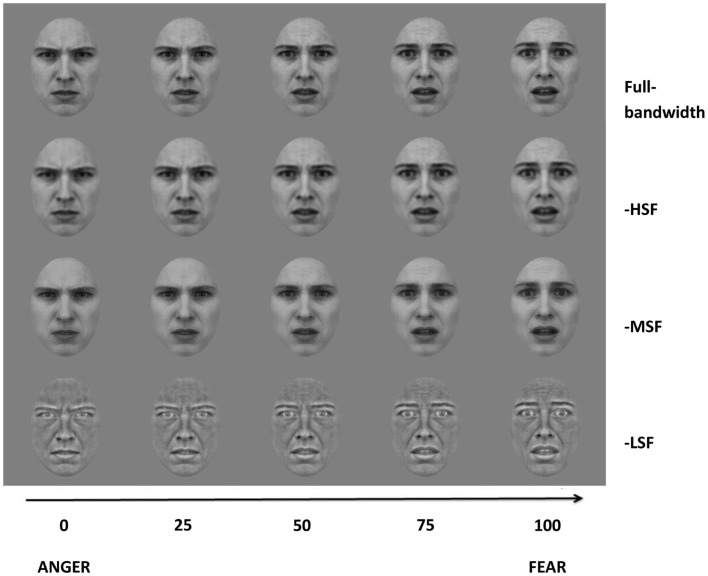
**Image examples from the four morph continua (Full-bandwidth, -HSF, -MSF, and -LSF)**. The abscissa represent the degree of morphing of the corresponding images.

A sequence of 101 images was created by morphing between the prototypical fear and anger expression face (Tiddeman et al., 2001), such that the 0 and 100 morph level corresponded to prototypical anger or fear, respectively, and the 50 morph level corresponded to a morph image with equal weighting of both expressions (Figure 1). Next, the set of morphed images was filtered to remove specific SF components of either low (<8 cycles/face), medium (12–28 cycles/face), or high (>32 cycles/face) bandwidth. This filtering procedure was conducted after the morphing procedure to minimize SF distortion (Tiddeman et al., 2001). Butterworth filters were applied to produce three sets of morphed continua: HF-subtracted condition, MF-subtracted condition, and LF-subtracted condition (Figure 1). Cut-off frequencies were 8 cycles/face, 12 and 28 cycles/face, and 32 cycles/face, respectively. Thus, the bandwidths of the low and high-pass filters are 3-octaves wide, while the middle-range notch-filter is approximately 1.2 octave-wide. The fourth-order Butterworth filters guaranteed a minimum of 4.3 dB attenuation before any frequency crossover occurred.

Next, an oval mask subtending 289 × 416 pixels was applied, producing an object area of 7.43° × 10.68° of visual angle. All images were presented at a mean luminance of 25 cd/m^2^. To standardize the presentation times of stimuli across all three experiments, an adaptation image of white noise, subtending an area of 13.1° × 13.1°, was displayed prior to the test images.

#### Procedure

**Figure 2:**
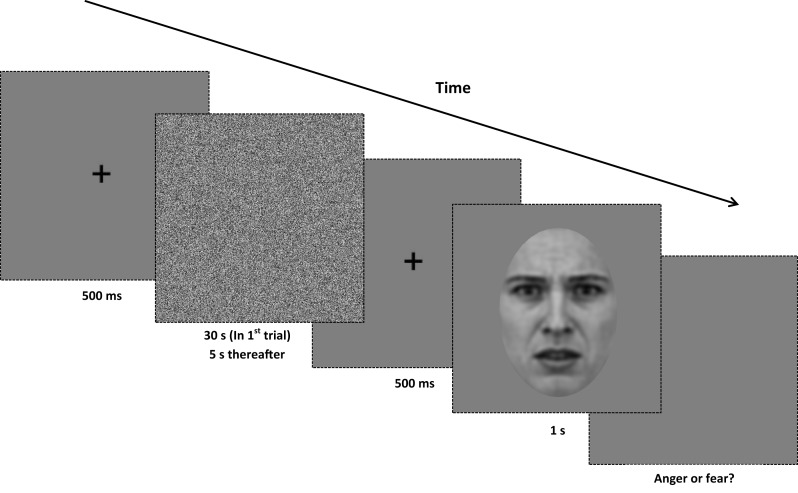
**Procedure for Experiment 1**. At the beginning of each trial, a central fixation point is presented for 500 ms. Next, a white noise adaptor is presented for 30 s in the first trial of the block, and for 5 s in every subsequent trial. After the noise stimulus, the central fixation point is displayed for another 500 ms. A test image (from either the full-bandwidth, -HSF, -MSF, or -LSF morph continua) is then displayed for 1 s, and the screen presents a gray background until a binary response (anger or fear) is made. All adaptors and test images are rotated in a randomized direction with a diameter of 1° of visual angle around the center of the screen.

In each trial subjects were required to classify the emotional expression of the test image by making a binary choice (“anger” or “fear”) on a standard keyboard. Subjects were required to maintain their attention on a white fixation point, which remained onscreen during the presentation of adaptation and test stimuli, the interstimulus interval and the response phase of the experiment. The white noise adaptor was presented for 30 s in the first trial and thereafter for 5 s duration intervals prior to the test image. After an interstimulus interval of 500 ms (containing a central fixation point only), the test image was displayed for 1 s (see Figure 2 for details). In the control sequence, a test image drawn from the unfiltered morph dataset was presented. In the experimental conditions (-HSF, -MSF, -LSF), a test image was drawn from one of the filtered morph ranges and presented. Test images appeared at a random location in a circular trajectory with a diameter of 1° of visual angle around the central fixation point and continued moving in a randomly determined directional rotation (clockwise or counter-clockwise) around the center of the screen. Subjects were instructed to maintain fixation on the cross location while the test image rotated in order to avoid retinotopic adaptation (Skinner and Benton, 2010).

After viewing the test image there was an interstimulus interval with the central fixation point only, and subjects were required to make a binary response classifying the expression in the image. Only once this response had been made, did the experiment progress to the next trial. The order of test image presentation was determined using Bayesian entropy estimation (Kontsevich and Tyler, 1999), updating previous probabilities in the estimated psychophysical function by selecting the morph level on each trial. The morph level in each trial was selected to yield the maximum expected information for prediction of the expected mean threshold.

The ocular distance to the screen was set to 80 cm. The order and design of the experimental blocks was based on a full Latin squares design. Prior to each experimental session, participants completed a training phase to minimize subsequent variability within the experiment. The training phase consisted of blocks using the control condition of full-bandwidth images. Participants only progressed to the experiment if the standard deviation of the last 10 thresholds in the block was lower than 3. In total, each participant completed 16 experimental blocks, spaced evenly into 4 experimental sessions, with 4 blocks per session. Each block contained a total of 40 trials. The block order and session order was pseudo-randomized.

Subjects’ responses for the 40 trials in each block were collected and threshold and slope parameters were estimated by fitting a Gaussian cumulative function. Each participant yielded a total of 16 thresholds. The Chi-square statistic of goodness-of-fit was used to assess the fit of the function and blocks containing a score of χ^2^ < −18 were discarded. The data from one participant was excluded from the analysis, due to lack of adequate fitting of their data (the majority of blocks were discarded).

All experimental procedures were conducted in accordance with the Conselho Nacional de Ética em Pesquisa rules and were approved by the Ethical Committee of the Federal University of ABC. An informed consent was obtained from all subjects.

### Results and discussion

**Figure 3:**
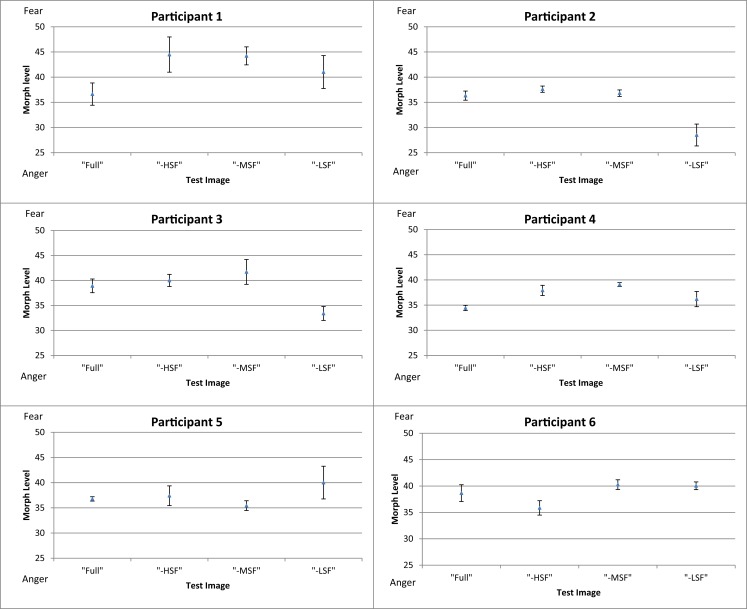
**Mean thresholds of morph level for the four conditions (full-bandwidth, -HSF, -MSF, and -LSF) for participants in Experiment 1**. Error bars show ±1 standard error of the mean.

Figure 3 shows the mean threshold results of the participants for each condition (full-bandwidth, -HSF, -MSF, and -LSF). For all subjects, the balance point between fear and anger in the full-bandwidth condition was at a morph level weighted toward anger (34–39). There was a relative shift toward anger for participants 1–4 when LF components were removed in comparison with the conditions in which medium or high-frequency components were removed. Thresholds for the middle- and high-frequency subtracted conditions were similar (participants 1–5) and/or shifted toward fear (participants 1, 2, and 4) in comparison with the control condition.

**Figure 4:**
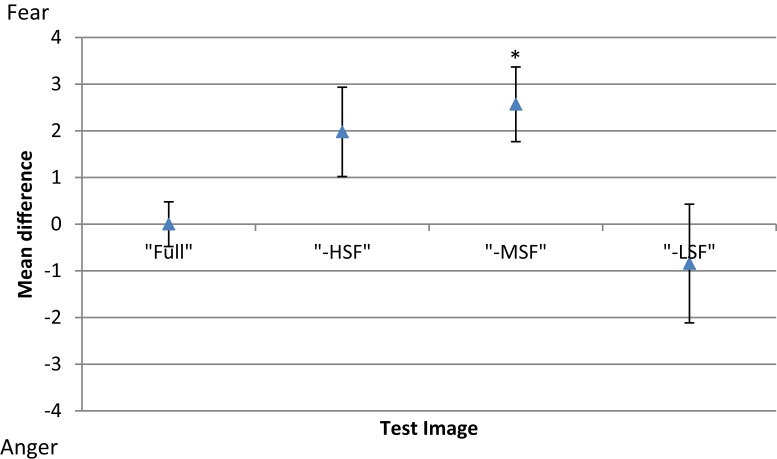
**Mean difference in threshold for each condition of test image type (full-bandwidth, -HSF, -MSF, and -LSF); with respect to the mean threshold of the control condition (full-bandwidth)**. Error bars show ±1 standard error of the mean. Asterisk represents significant difference at *p* < 0.05 level from full-bandwidth condition.

To investigate the between-subject effect of the different conditions, the threshold of each participant for each experimental condition was subtracted from his mean threshold of the control condition (Figure 4). Results show that removing high or middle-frequency components shifts the balance point toward fear, i.e., without these components, there is an increase in responses classifying the expression as anger in comparison to the control condition. The opposite effect is observed when low frequency components are removed from the face images, where there is an increase in the reported perception of fear.

A one-way repeated-measures ANOVA was conducted to determine statistical significance of the differences in thresholds. There was a significant main effect of filtering condition [*F*(3, 69) = 4.423, *p* < 0.01]. *Post hoc* tests using the Dunnett correction revealed a significant difference between the -MSF (*M* = 39.59, σ_M_ = 0.688) and full-bandwidth condition (*M* = 36.96, σ_M_ = 0.688), *p* < 0.05. All other differences between conditions were non-significant (*p* > 0.05).

The results indicate that middle-frequency spatial information is more critical for anger encoding than for fear, while high-frequency components might also dominate anger representation. Considering the literature reviewed in the Section “Introduction,” by itself, this result reflects processing at low-level facial expression processing, high-level, or both. In selecting between angry and fearful expressions, SF information may be flexibly used (as suggested by Schyns and Oliva, 1999), where the higher level of threat associated with anger is preferentially encoded through low SF information, while medium and high SF information is recruited for the perception of fear. To test the effect of SF information at a higher level of processing facial expressions, an adaptation paradigm was adopted and forms the basis of Experiment 2 and 3.

## Experiment 2: Adaptation to Filtered Expressions of Fear

### Materials and methods

Experiment 2 shares partially the methods already described in Experiment 1 and will be described only briefly. Five volunteers from the Federal University of ABC (two male and two female between 18 and 29 years of age) with normal or corrected-to-normal vision participated in the experiment. Only subjects who had not participated in Experiment 1 were included in Experiment 2. The apparatus used was identical to that used in Experiment 1.

A 100-level morph stimulus (prototypical fear) was used as the template for all adaptation stimuli. To test the combined adaptation effect of the SF magnitude and the phase component present in the expression, four adaptation images were generated by Fourier transforming the 100-level morph image. Images were then either filtered through a low-pass, band-pass, or high-pass Butterworth filter, or left unfiltered. This operation produced four adaptor stimuli: low SF (<8 cycle/image), medium SF (12–28 cycles/image), high SF (> 32 cycles/image), and full-bandwidth SF fear images. A white noise control adaptor was used as a baseline condition. Images from the full-bandwidth morph range, described in Experiment 1, were used as test images. All adaptation and test images had a size of 7.43° × 10.7°.

**Figure 5:**
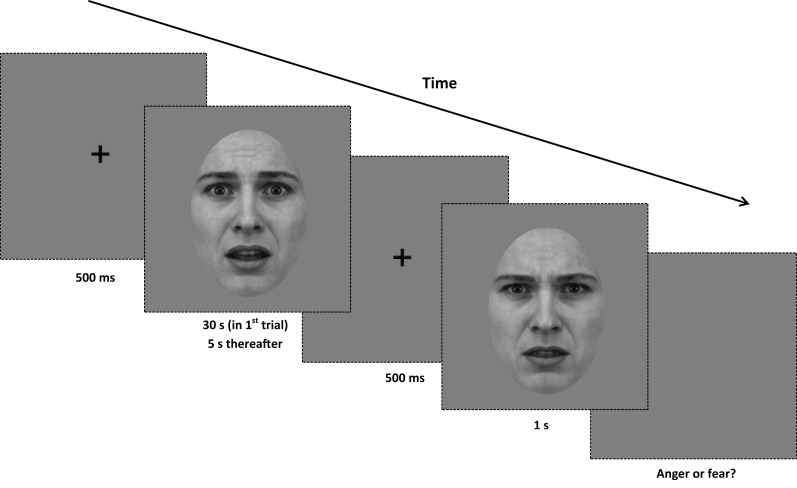
**Procedure for Experiment 2**. At the beginning of each trial, a central fixation point is presented for 500 ms. Next, an unfiltered or filtered face adaptor is presented for 30 s in the first trial of the block, and for 5 s in every subsequent trial. After the noise stimulus, the central fixation point is displayed for another 500 ms. A test image (from the full-bandwidth morph range) is then displayed for 1 s, and the screen presents a gray background until a binary response (anger or fear) is made. All adaptors and test images are rotated in a randomized direction with a diameter of 1° of visual angle around the center of the screen.

Experimental instructions, stimuli presentation intervals, response collection, and the method of threshold estimation were identical to those of Experiment 1. In this experiment only the adaptor and test images were altered. There were five adaptation conditions: white noise, LF fear, MF fear, HF fear, and full-bandwidth fear. Forty full-bandwidth test images were used (Figure 5).

The procedure and design of the training phase was identical to that used in Experiment 1. The order and design of blocks were based on a partial Latin squares design. In total, each participant completed 12 experimental blocks, spaced evenly into three experimental sessions, with four blocks per session. Each block contained a total of 40 trials. The block order and session order was pseudo-randomized for all participants. The data from one participant was excluded from the analysis, due to lack of adequate fitting of their data (the majority of blocks were discarded).

### Results and discussion

**Figure 6:**
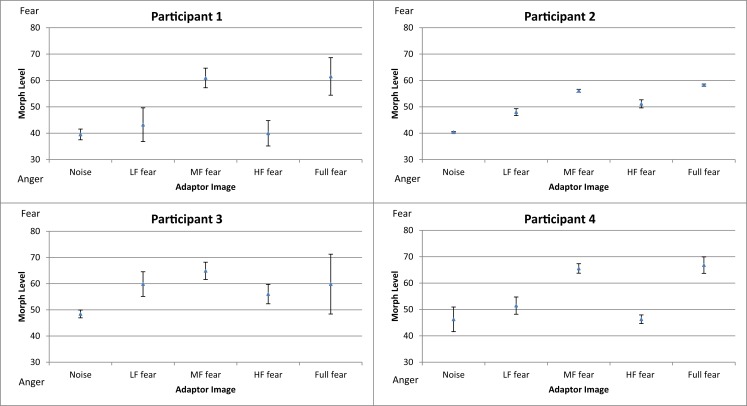
**Mean thresholds of morph level for the five conditions (white noise, LF fear, MF fear, HF fear, and full-bandwidth fear) for the participants in Experiment 3**. Error bars show ±1 standard error of the mean.

Figure 6 shows the mean threshold results of the participants for each condition (white noise, LF fear, MF fear, HF fear, and full-bandwidth fear). Adaptation to the MF fear and full fear conditions produces consistently stronger aftereffects than adaptation to the LF fear and HF fear conditions. All subjects show adaptation effects for the MF and full-bandwidth fear conditions compared with the control condition. Participants 2 and 3 also show adaptation effects for the LF and HF fear conditions. To calculate a measure of the adaptation strength for all condition, thresholds were subtracted from the mean threshold of the control condition, producing mean difference scores representing adaptation strength (Figure 9).

A one-way repeated-measures ANOVA was conducted to determine statistical significance of threshold difference. There was a significant main effect of adaptation condition [*F*(4, 24) = 16.360, *p* < 0.01]. *Post hoc* tests using the Dunnett correction revealed a significant difference between white noise condition (*M* = 43.65, σ_M_ = 5.57) and LF fear condition (*M* = 50.63, σ_M_ = 9.02), *p* < 0.05, MF fear condition (*M* = 61.60, σ_M_ = 5.36), *p* < 0.01, and full-bandwidth fear condition (*M* = 61.79, σ_M_ = 7.77), *p* < 0.01. HF fear condition (*M* = 48.35, σ_M_ = 7.83) was not significantly different from the white noise condition (*p* > 0.05).

The full-bandwidth fear adaptor shifted perception toward anger, a result that corroborates the literature by suggesting a polarity between anger and fear face processing, but which does not suffice to answer whether it is related to the SF content or not. The results of the other conditions further indicate that the adaptation effect is specific to low- and middle-frequency components, but not high-frequency. In consideration of the literature presented in the Section “Introduction,” it can be argued that these results reflect characteristics of high-level processing. To test the adaptation effect of facial expression without phase information, we conducted Experiment 3.

## Experiment 3: Adaptation to Filtered Expressions of Fear at Random-Phase

### Materials and methods

Experiment 3 shares partially the methods already described in Experiment 1 and will be described only briefly. Six volunteers from the Federal University of ABC (all male between 18 and 45 years of age) with normal or corrected-to-normal vision participated in the experiment, including authors YZ and WC (participants 5 and 6, respectively). Only subjects who had not participated in Experiment 1 and 2 were included. The apparatus used was identical to that of Experiment 1.

A 100-level morph stimulus (prototypical fear) was used as the template for all adaptation stimuli (same as in Experiment 2). To test adaptation effects related to specific SF bandwidths, four adaptors were generated by first Fourier transforming the images. Images were then either filtered through a low-pass, band-pass, or high-pass Butterworth filter, or left unfiltered. Next, the phase part of the frequency components was randomized and an inverse Fourier transformation was applied (Hansen et al., 2008). This operation produced four adaptor stimuli: low SF (0–8 cycle/image), medium SF (12–28 cycles/image), high SF (>32 cycles/image), and full-bandwidth SF random-phase images. A white noise control adaptor was used as a baseline condition. Images from the full-bandwidth morph range, described in Experiment 1, were used as test images. Adaptation images subtended over an object area of 13.1° × 13.1°, while all test images spanned 7.43° × 10.7°.

**Figure 7:**
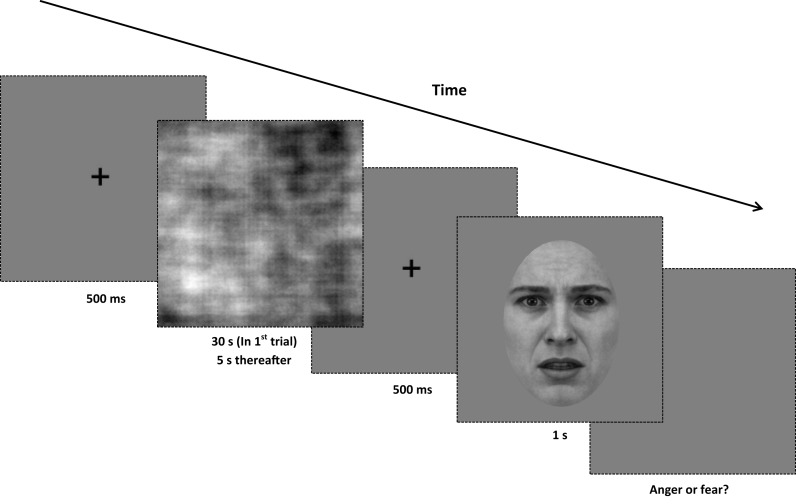
**Procedure for Experiment 3**. At the beginning of each trial, a central fixation point is presented for 500 ms. Next, a random-phase adaptor is presented for 30 s in the first trial of the block, and for 5 s in every subsequent trial. After the adapting stimulus, the central fixation point is displayed for another 500 ms. The test image (from the full-bandwidth morph range) is displayed for 1 s, and the screen presents a gray background until a binary response (anger or fear) is made. All adaptors and test images are rotated in a randomized direction with a diameter of 1° of visual angle around the center of the screen.

Experimental instructions, stimuli presentation intervals, response collection, and the method of threshold estimation were identical to those of Experiment 1. In this experiment only the adaptor and test images were altered. There were five adaptation conditions: white noise, LF random-phase, MF random-phase, HF random-phase, and full-bandwidth random-phase. Forty full-bandwidth test images were used (Figure 7).

The procedure and design of the training phase were identical to that used in Experiment 1. The order and design of blocks were based on a partial Latin squares design. Each participant completed 15 experimental blocks, spaced evenly into three experimental sessions, with five blocks per session. Each block contained a total of 40 trials. The block order and session order were pseudo-randomized for all participants.

### Results and discussion

**Figure 8:**
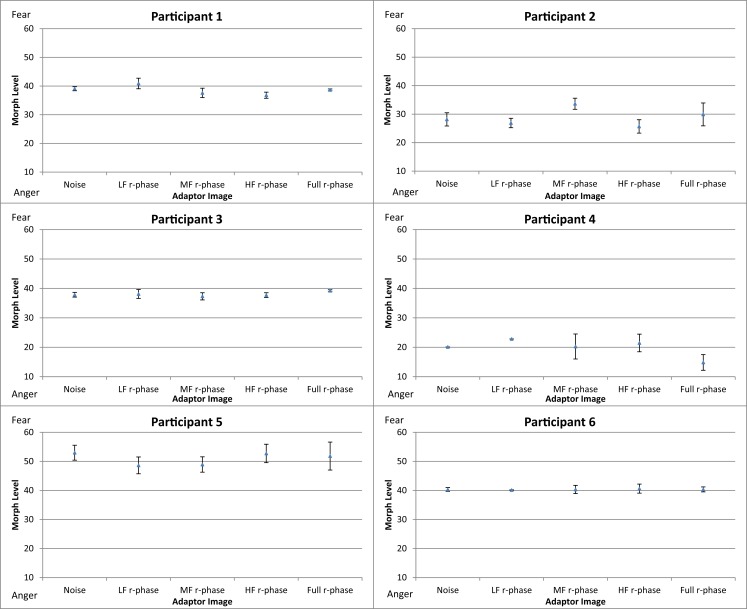
**Mean thresholds of morph level for the five conditions (white noise, LF random-phase, MF random-phase, HF random-phase, and full-bandwidth random-phase) for participants in Experiment 3**. Error bars show ±1 standard error of the mean.

Figure 8 shows the mean threshold results of the participants for each condition (white noise, LF random-phase, MF random-phase, HF random-phase, and full-bandwidth random-phase). There appear to be no consistent differences between the control condition (white noise) and the adaptor conditions. The balance point varied strongly between participants, in the 20–50 range. That variation probably represents differences in criteria level, while the main effect is calculated via the deviation from the control condition.

**Figure 9:**
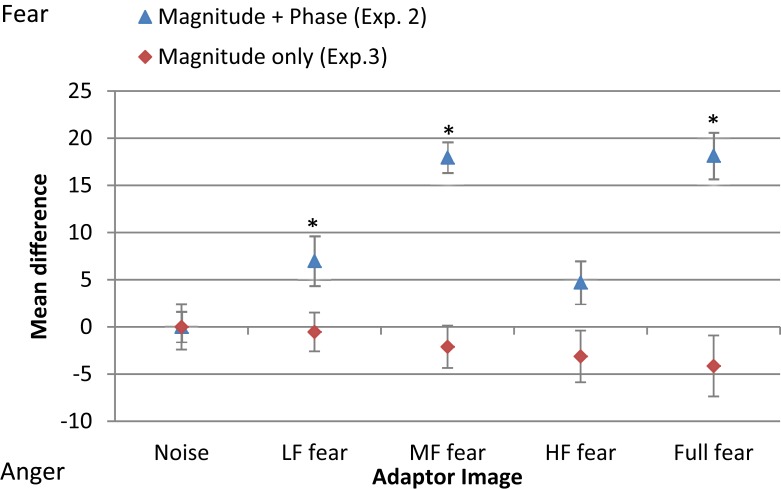
**Mean difference in threshold for each condition of adaptor image (white noise, LF fear, MF fear, HF fear, full-bandwidth fear) with respect to the mean threshold of the control condition (white noise)**. Blue triangles represent results from adaptor images with magnitude and phase spectra (Experiment 2). Red diamonds represent results from adaptors with phase spectrum only (Experiment 3). Error bars show ±1 standard error of the mean. Asterisks represent significant difference at *p* < 0.05 level from white noise condition.

To compare the difference in threshold shift between the control condition (white noise) and the experimental conditions (LF random-phase, MF random-phase, HF random-phase, full-bandwidth random-phase) the thresholds for each condition were subtracted from the mean threshold of the control condition, producing mean difference scores for every condition (Figure 9). A one-way repeated-measures ANOVA was conducted, revealing no significant main effect of adaptation condition, *F*(4, 55) = 0.003, *p* > 0.05.

The absence of significant differences between the balance-points at different adaptation conditions indicates that the information content of the phase spectrum is critical for encoding fear and anger expressions.

## General Discussion

The experiments reported in this study examined the effect of specific SF bandwidth manipulation on the classification of facial expressions under two alternative paradigms: SF subtraction and SF adaptation. Shifts along the anger-fear dimension as a result of subtracting specific SF components can indicate the critical information used for representing the expressions. Here we show that subtracting medium-frequency components significantly shifts the balance point toward the fear prototype in comparison to the control condition, indicating that these components are used for encoding fear more than for anger. As previously suggested, different SF ranges may be recruited when presenting both expressions simultaneously as opposed to presenting either expression in isolation. Accordingly, the lesser degree of threat present in fear may result in increased recruitment of medium SF channels, and not low SF channels. Subtracting low frequency components, on the other hand, did not produce a significant shift of the balance point, suggesting that they are equally critical for anger and fear classification. However, although the results are significant, the effect is weak, indicating that anger and fear classification can rely, although with partial accuracy, on any SF bandwidth.

Previous studies indicate that this paradigm predominantly probes low-level processing of face stimuli (Halit et al., 2006; Flevaris et al., 2008), consequently these results suggest that fear encoding, in comparison with anger, depends more on medium-frequency sources at the initial processing stages. To probe higher-level processing, an adaptation approach was taken. The results (Figure 9) show that adaptation to full-spectrum fear shifted the balance point toward the fear prototype, indicating a relative increase in sensitivity to perception of anger. This result is in accordance with previous studies (Webster et al., 2004; Juricevic and Webster, 2012). Experiment 2 investigated the specific spatial components associated with this effect, revealing that low- and middle-frequency components, with a predominance of the latter, are the most influential when processing fearful face images. Thus, middle-frequency components seem to be the most critical for fear encoding at both low and high-level processing. However, the adaptation effect of filtered images in Experiment 2 could be due to the effect of (1) low-level SF content, (2) high-level phase information, or (3) both. Experiment 3 was designed to exclude any featural and/or configural information present in the phase spectrum, leaving untouched the magnitude of the frequency components by randomizing the phase spectrum. The results indicate that the adaptation effect observed in Experiment 2 is dependent on the phase information. As a whole, the results suggest that (1) mid frequency components are the most critical for encoding the fearful facial expression on the anger-fear axis at both low- and high level of processing and that (2) high-level processing of fear relies more on facial information content present in the phase spectrum which may include featural and configural information.

The adaptation technique used in the present study to investigate the effect of different SFs on face representations has visible antecedents: Vuilleumier et al. (2003) discovered an asymmetrical habituation effect in the fusiform gyrus. Participants habituated to the same face identity when the facial image was shown first in a high-pass version and later as a low-pass version but not vice versa. If a neural face representation were independent of the type of SF information processed, we would expect to see a symmetrical habituation effect instead (Gauthier et al., 2005). Kumar and Srinivasan (2011) recently examined the effect of SF specificity on reaction time and error rate in the discrimination of facial expressions of happiness and sadness. They concluded that low SF bandwidths (<8 cycles/face) were diagnostic for classification of happy expressions, while high SF bandwidths (>32 cycles/face) were critical for processing of sad expressions. However their dependent measures, used also in other studies (Deruelle and Fagot, 2004; Aguado et al., 2010), give no indication as to the level of SF processing. The limitations of calculating a “critical bandwidth” for processing individual facial expressions are becoming increasingly clear, as can be seen from the findings that SF information is flexibly used depending on the task type (Schyns and Oliva, 1999) and/or viewing distance (Smith and Schyns, 2009).

Visual channels within the brain appear to be tuned to specific SF bandwidths, varying by octave (e.g., 2–4, 32–64), and correspond to changes along a contrast sensitivity function (Ruiz-Soler and Beltran, 2006). Low and high SF information is processed in parallel through separate neural pathways, with fast, unconscious processing of coarse low SF information occurring through the pulvinar, superior colliculi, amygdala and other subcortical structures, and slower, more refined analysis of detailed high SF information occurring through cortical visual areas including the fusiform gyrus (Vuilleumier et al., 2003; Rotshtein et al., 2007). Sowden and Schyns (2006) have argued that flexible use of diagnostic SF information depends on top-down selection between different SF channels which may be a product of learning the relevance of available SF content, the viewing environment and task-based expectations. The evidence for top-down selection of SF channels is inconclusive: studies of SF uncertainty show improved performance on blocks where the target SF is the same as opposed to intercalated with other SFs. Moreover, these uncertainty effects on intermixed blocks are reduced or suppressed if the target SF is cued by a sound or number (Davis et al., 1983; Hübner, 1996), yet critical-band noise-masking studies show no evidence of top-down selection between SF channels (Solomon and Pelli, 1994; Lu and Dosher, 2004; Talgar et al., 2004). Our results indicate that SF information is processed alongside other diagnostic facial information to show selectivity in the higher-level representation of facial stimuli. This conclusion offers some clarification to the inconclusive findings of top-down selection of SF channels and goes some way to identifying at which stage SF and other forms of visual information are integrated in facial processing.

This study offers a timely and effective means of bridging face adaptation and SF research. Future research in this area should go beyond determining “critical values” for identity and expression, and investigate how specific SF bandwidths are integrated through multiple channels and at different stages of processing, how selection of SF information changes in relation to the scarcity of visual input or noise distraction and the role of learning in the selection of diagnostic or relevant information in response to task stimuli.

## Conflict of Interest Statement

The authors declare that the research was conducted in the absence of any commercial or financial relationships that could be construed as a potential conflict of interest.
